# Duration of solid fuel cookstove use is associated with increased risk of acute lower respiratory infection among children under six months in rural central India

**DOI:** 10.1371/journal.pone.0224374

**Published:** 2019-10-24

**Authors:** Lauren Arlington, Archana B. Patel, Elizabeth Simmons, Kunal Kurhe, Amber Prakash, Sowmya R. Rao, Patricia L. Hibberd

**Affiliations:** 1 Department of Global Health, Boston University School of Public Health, Boston, Massachusetts, United States of America; 2 Lata Medical Research Foundation, Nagpur, Maharashtra, India; 3 Department of Pediatrics, Indira Gandhi Government Medical College, Nagpur, Maharashtra, India; Valahia University of Targoviste, ROMANIA

## Abstract

**Introduction:**

India has a higher number of deaths due to acute lower respiratory infections (ALRIs) in children <5 years than any other country. The underlying cause of half of ALRI deaths is household air pollution from burning of solid fuels, according to the World Health Organization. If there is a direct association between duration of exposure and increased ALRI risk, a potential strategy might be to limit the child’s exposure to burning solid fuel.

**Methods and materials:**

Children born to pregnant women participating in the Global Network for Women and Children's Health Maternal and Newborn Health Registry near Nagpur, India were followed every two weeks from birth to six months to diagnose ALRI. The number of hours per day that the child’s mother spent in front of a burning solid fuel cookstove was recorded. Children of mothers using only clean cookstoves were classified as having zero hours of exposure. Odds Ratios with 95% confidence intervals were obtained from Generalized Estimating Equations logistic models that assessed the relationship of exposure to solid fuels with risk of ≥1 ALRI, adjusted for sex of the child, household smoking, wealth, maternal age, birth weight and parity.

**Results:**

Between August 2013 and March 2014, 302 of 1,586 children (19%) had ≥1 episode of ALRI. Results from the multivariable analysis indicate that the odds of ALRI significantly increased from 1.2 (95% CI: 0.7–2.2) for <1 hour of exposure to 2.1 (95% CI: 1.4–3.3) for >3 hours of exposure to solid fuel cookstoves compared with no exposure (p<0.01). Additionally, decreasing wealth [middle: 1.2 (0.9, 1.6); poor: 1.4 (1.2–1.7); p<0.001] was associated with ALRIs.

**Conclusions:**

Our study findings indicate that increasing the time mothers spend cooking near solid fuel cookstoves while children are in the house may be associated with development of ≥1 ALRI in children <6 months.

## Introduction

Acute lower respiratory infections (ALRIs), predominantly pneumonia, account for more deaths among children under-five than any other infectious disease [1]. India has almost three times the number of ALRI deaths of any other country [2]. Vaccination is an important tactic to reduce childhood bacterial pneumonia and the conjugate pneumococcal and *Hemophilus influenzae* B vaccinations are increasingly available to children in low- and lower-middle-income countries [3,4]. Both vaccines are recommended in India [5], but the conjugate pneumococcal vaccine was not available outside the private sector until May 2017 [6]. While the immune response to the vaccines are developing in the first six months of life [7], there is an important role for other strategies to prevent ALRIs.

Household air pollution (HAP) from cooking and heating the home with solid fuels such as wood, crop waste, coal and animal dung on open fires or inefficient cookstoves is responsible for more disease and illness than any other environmental risk factor worldwide [7], including India [8]. The inefficient burning of solid fuels releases harmful levels of pollutants, including fine particulate matter [9]. Fine particulate matter, or PM_2.5_, can travel deep into the lungs and enter the blood stream when inhaled leading to increased risk for respiratory and cardiac diseases [10]. The World Health Organization set the 24-hour standard for mean concentration of fine particulate matter in the ambient air at 25 μg/m^3^[11]. Rural households burning solid fuels can reach mean daily PM_2.5_ concentrations of 609 μg/m^3^ in the kitchen [12]. Women and children have the highest personal exposures to HAP because of the time they spend inside the house and in the proximity of cookstoves [12]. HAP exposure due to cooking from solid fuels increases the risk for ALRIs among children [13,14], although a recent meta-analysis reported no benefit of improved cookstoves on ALRIs either [15]. In India, there is evidence that exposure to HAP increases the incidence of life-threatening respiratory illnesses, such as ALRIs, in children under the age of six [16]. While no evidence exists that quantifies the risk between duration of exposure to HAP and ALRI incidence within the Indian context, this relationship has been observed in Sierra Leone [17].

The critical need to reduce HAP has been recognized in recent years and was reaffirmed by the 2018 Global Burden of Disease Study [18]. The Global Alliance for Clean Cookstoves is leading the effort in driving adoption of clean cookstoves and fuel. However, rollout and acceptance of clean cooking alternatives is progressing slowly [19]. The Indian Government’s initiative Pradhan Mantri Ujjwala Yojina, launched in May 2016, is already providing access to clean cooking gas for about 31 million of the 50 million households below the poverty line [20]. However, there are an additional 50 million households in India still using solid fuel and the children living there are at risk of death from ALRI. If there is a direct association between duration of exposure to pollutants from use of solid fuel and risk of developing ALRI, potential interim strategies need to be considered such as educating the mothers to limit the child’s exposure to burning solid fuel, reducing the number of hours of cooking time or encouraging other household members to care for the infant away from the stove while the mother is cooking.

The *Eunice Kennedy Shriver* National Institute of Child Health and Human Development’s Global Network for Women and Children’s Health Research supports a Maternal and Newborn Health (MNH) Registry of pregnant women and their babies living in rural communities in six low- and lower-middle-income countries [21]. In the Nagpur site in India, we have the Institutional Review Board approval to follow the infants born to women enrolled in the Registry for six months after birth. Therefore, the Nagpur site of the Registry provides an ideal population to address questions about risk factors for ALRI in rural communities where about two thirds of households use solid fuels [22]. We hypothesized that the number of hours per day a child’s mother spent in front of a lit or smoldering solid fuel cookstove would be an easily measurable proxy for duration of exposure of the child to HAP as the child is often in the house while the mother is cooking [17]. The aim of the HAP-ALRI add-on study to the MNH Registry is to determine whether there is an association between duration of time in front of burning solid fuel and ALRI in the rural villages near Nagpur, India.

## Materials and methods

### Study design and setting

The HAP-ALRI add-on study was a prospective observational study that enrolled children born to women participating in the Global Network for Women’s and Children’s Health Research MNH Registry Study (registered on clinicaltrials.gov under NCT01073475) in Nagpur, India and followed them from birth through 6 months of age. Nagpur is approximately 310 meters above sea-level and there is little variation in altitude between study villages. Nagpur has an ALRI season that runs from October through March during which temperatures are cooler, averaging 68–83°F (20–28°C), and there is less rainfall compared to the rest of the year.

**Fig 1:**
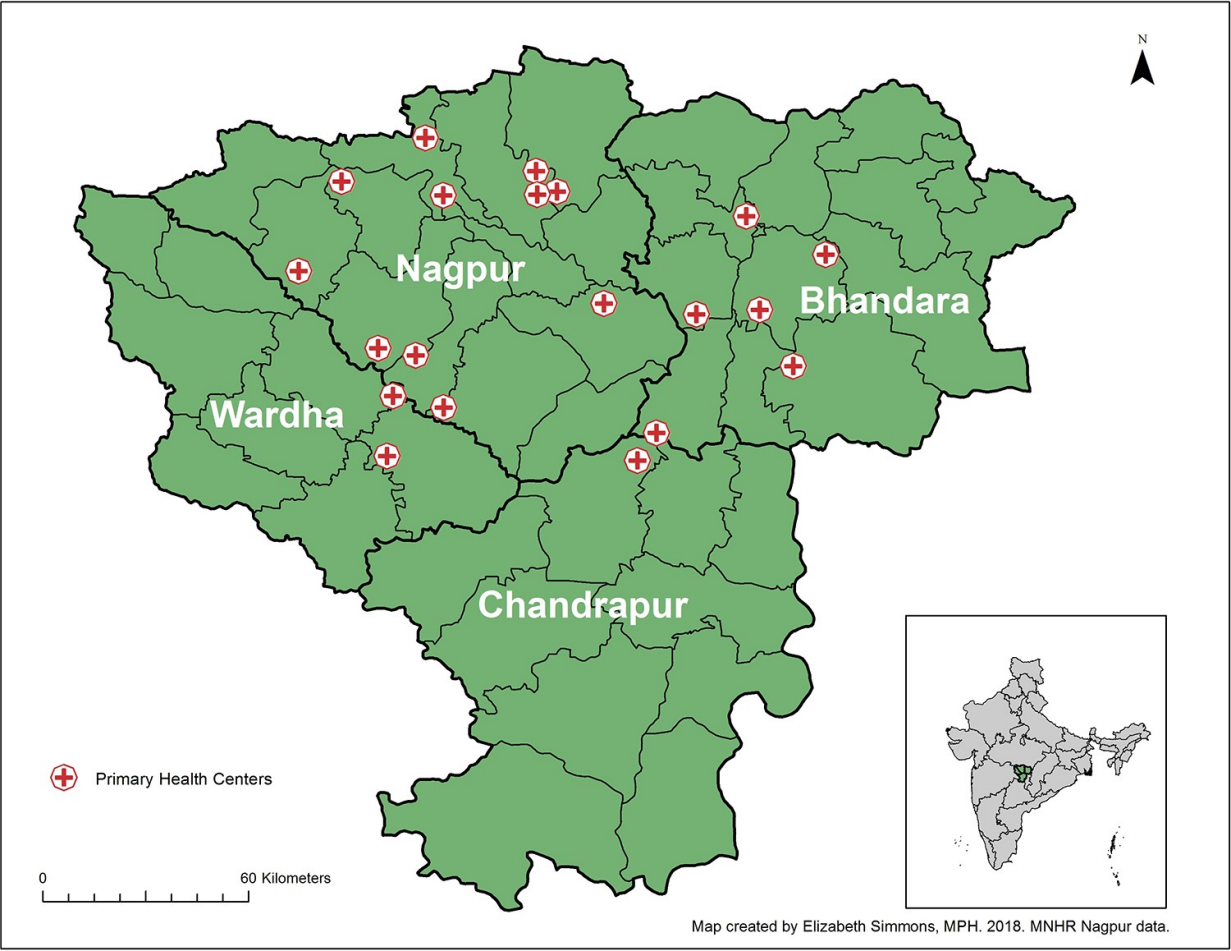
Four districts in Nagpur and locations of primary health centers.

The details of the MNH Registry Study can be found elsewhere [21]. Briefly, women are enrolled in the Registry as early as possible in their pregnancy. In the Nagpur site, the women are recruited from villages in the neighboring areas of 20 rural Primary Health Centers (PHCs) that served a population of approximately 536,000 in 2010 (Fig 1). Women are followed through labor and delivery and until 42 days postpartum to collect morbidity and mortality data on the mother and newborn using MNH data collection forms.

### Ethical considerations

Written informed consent from each mother was obtained in Marathi, the local language. Approval for this study was obtained from the Institutional Review Boards at Lata Medical Research Foundation and the Partners Human Research Committee.

### Data collection

Data were collected between September 4, 2013 and July 1, 2014. For the HAP-ALRI add-on study we used three forms (HAP01, HAP02 and HAP03) to collect the data that were not available in the MNH Registry. HAP01 (baseline) and HAP02 were collected within two weeks of the child’s birth, and HAP03 and the daily diary were collected every two weeks throughout the first six months of the child’s life. HAP01 was used to collect information on household characteristics, maternal characteristics and cooking practices of the household. HAP02 was used to collect information on cooking practices and smoking habits of that household from women who spent at least one month of pregnancy living at a house different from their current house of residence. HAP03 was used to collect information on the child’s morbidity, mortality, and breastfeeding habits. If a household stove was in use during the two weeks prior to the survey, the location of the stove was also collected in HAP03. Questions pertaining to information on ALRI episodes were adapted from the Phase 6 Women’s Questionnaire of the Demographic Health Survey [23]. The episodes were self-reported by the child’s mother on the HAP03 questionnaire at their home visit every other week and included any episode occurring in the two weeks prior to each visit. To improve recall of children’s symptoms, mothers were requested to complete a pictorial diary in which they placed a “bindi” or red dot sticker on the days that children had each of the following symptoms: fever, cough, difficulty breathing, runny nose, diarrhea, and blood in stool. Use of the pictures in the diary allowed all mothers to use the form, regardless of literacy. All questionnaires were field tested prior to data collection.

Accredited Social Health Activists (ASHAs), community health workers instituted by the government and living in the villages they serve, collected all the data and the diaries completed by the mother. The ASHAs were trained centrally by the MNH Registry staff on how to complete the forms. The ASHAs also provided information to the mothers about completing the daily pictorial diary regarding the symptoms of illness in their child. Hence, they were shown training videos of babies with symptoms of ALRIs, including difficulty breathing and chest wall in-drawing, in addition to their routine government trainings on recognizing maternal and childhood illnesses in the communities they serve. However, they were not trained to look for ALRIs in houses with a specific type of stove. Registry staff held monthly meetings with the ASHAs to collect and review the completed forms and answer any questions about the conduct of the study.

### Exposure

Duration of exposure to HAP was collected on the HAP01 and HAP02 (if applicable) questionnaires and defined by the usual number of hours per day the child’s mother spent in front of a solid fuel cookstove while it was lit or smoldering. Solid fuel cookstoves were chulhas (handmade, three-sided earthen stoves with open flames in the center) or open fires burning wood, straw, shrubs, grass, agricultural crop, animal dung, coal or charcoal. Exposure information was collected on the stove in the household the mother identified as being used most often for cooking (primary stove). Households using only clean fuels for their primary stove (liquid petroleum gas or electricity) were classified as having zero hours of exposure to solid fuel. Duration of exposure to solid fuel was categorized as 0, ≤1, >1 and ≤2, >2 and ≤3, and >3 hours. Other studies in India have shown that over 50% of children ages 5 years and under are with their mothers during cooking activities, so we infer that the mother’s exposure is an appropriate proxy for the child’s exposure [24].

### Primary outcome

The primary outcome is any episode of ALRI, defined as the presence of cough accompanied by difficulty breathing (fast breathing with short, rapid breaths or chest wall in-drawing) [25]. Children are categorized as either having or not having ALRI during the study period.

### Covariates

Covariates included in the analysis are grouped into three categories: (1) child-level factors from the MNH Registry: sex (male, female) and birth weight (≥2.5 kg and <2.5 kg); (2) maternal-level factors from the MNH Registry: age in years (<25, ≥25) and parity (nulliparous, multiparous); and (3) household-level factors: smoking in the household (yes, no) and wealth (rich, middle, poor). For women who spent more than one month away from their primary residence during their pregnancy, household-level factors were collected for the secondary residence as well. To calculate wealth, we adapted methodology from the Demographic and Health Surveys (DHS) using principal components analysis to create wealth quintiles measuring socioeconomic status [26]. The composite variable used indicators of household wealth collected on HAP01 including building material of the floor, roof, and walls of the house, land and animal possession, crowding, household assets, toilet facilities, and water supply. Unlike many other surveys following this methodology, cooking fuel was not included in the creation of our wealth variable as it is directly related to the exposure. The two lowest and two highest groups were combined to create a 3-level wealth variable (rich, middle, poor) to increase the robustness of our analysis.

### Statistical methods

Summary measures (proportions) were obtained for all variables. Bivariate analyses tested the significance of the association of the variables with presence or absence of any ALRI using a two-sided Chi-square test of independence.

Regression analyses were conducted to identify risk factors of ALRI and adjusted for clustering of children within PHC. Twenty PHCs are included in this analysis, but four with <60 children represented in this study are grouped together into two clusters of two each in order to have large enough samples to produce stable estimates. We obtained adjusted odds ratios (ORs) with 95% confidence intervals (CIs) from a multivariable Generalized Estimating Equations model to evaluate the association of duration of exposure to HAP with having ≥1 ALRI adjusting for all covariates described above. All covariates were considered as potential confounders, a priori. Since we were concerned that the interaction of smoking, birth weight and wealth with exposure might affect the relationship of the exposure with ALRI, we tested them in the regression models. All analyses were conducted on complete data using SAS 9.4 (SAS Institute, Cary, NC) and a two-sided p<0.05 was considered to be statistically significant.

## Results

### Study population

**Fig 2:**
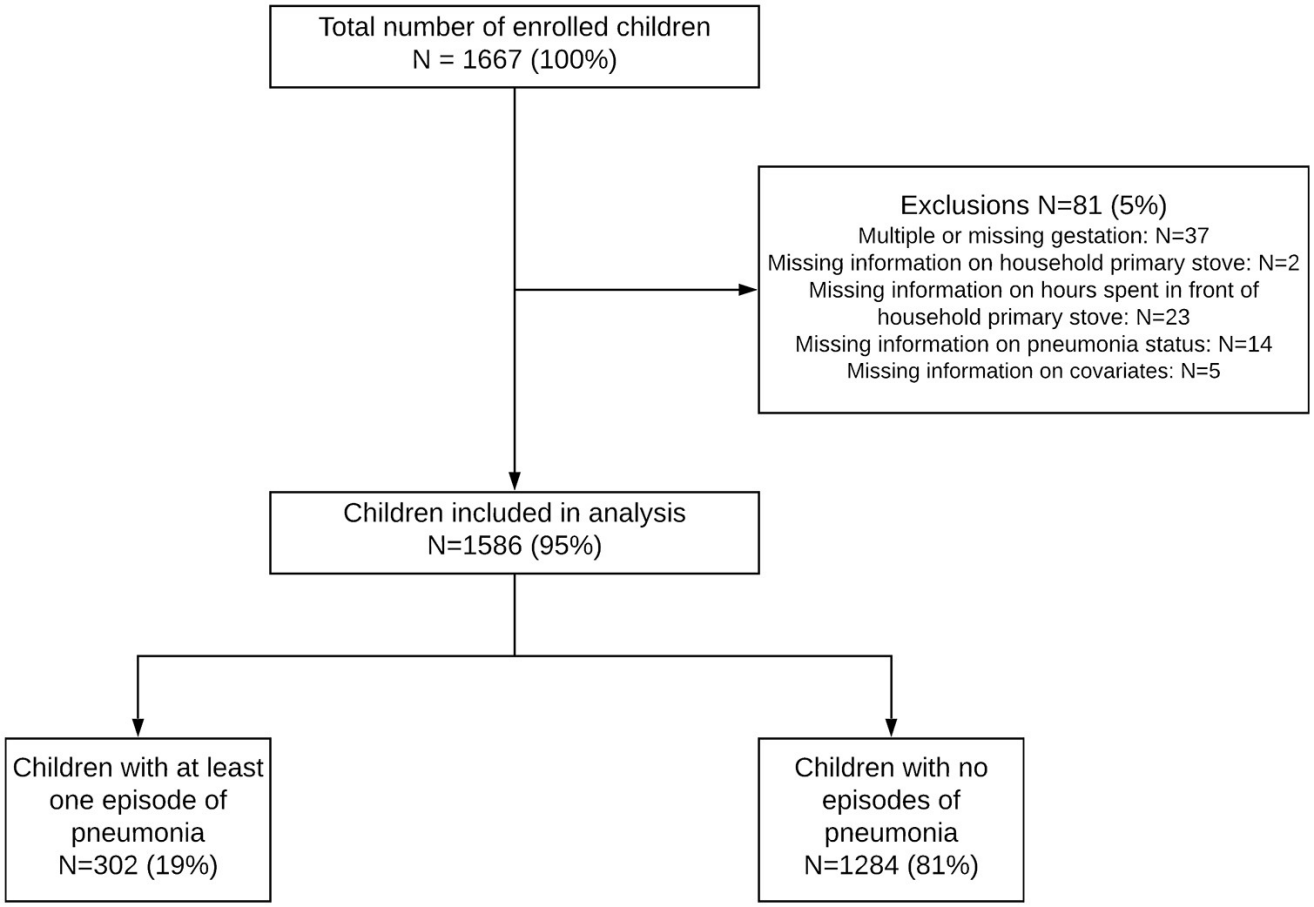
Study population flow diagram.

The study population consisted of babies born between August 20, 2013 and March 2, 2014 to mothers enrolled in the MNH Registry in Nagpur, India. Of the 1,667 children enrolled in the study, 81 were excluded due to multiple or missing gestation or missing information on household primary stove, hours spent in front of solid fuel cookstove, ALRI status or covariates (Fig 2). Of the remaining 1,586 children, 302 (19%) experienced at least one ALRI episode during the study while 1,284 children experienced no episodes.

Overall, 107 (6%) of the households used only clean cookstoves, 930 (56%) used only polluting cookstoves and 628 (38%) used a mixture of clean and polluting cookstoves (stove stacking). Of those using a mixture of clean and polluting cookstoves, 63% considered the clean stove their primary stove and 37% considered the polluting cookstove their primary stove.

Only 6% (n = 96) of our population moved to another residence for at least 30 days during their pregnancy. These numbers are not sufficient to conduct a secondary analysis. For the women who moved, the distribution of our exposure variable is similar between residences (S1 Table).

Table 1 displays the distribution of variables by outcome. Compared with children with no episodes of ALRI, children with ≥1 ALRI were *less likely* to live in homes with a clean cookstove (21.8% vs. 33.7%; p<0.001) and be in the rich wealth category (32.1% vs. 42.4%; p<0.01), and *more likely* to live in homes with smokers (18.9% vs. 14.1%; p = 0.04) or be born to mothers aged <25 (67.9% vs. 59.6%; p<0.01). Sex of the child, birth weight and parity of their mother are similarly distributed in the two groups.

**Table 1:** Background characteristics of 1586 children followed from August 20, 2013 to March 2, 2014.

	Episodes of ALRI		
	None (n = 1284)	At least 1 (n = 302)	
	N (Col %)		P-value^a^
**Hours spent in front of polluting cookstove (per day)**			
No polluting cookstove	433 (33.7)	66 (21.8)	<0.001
≤1 hour	117 (9.1)	26 (8.6)	
1< hours ≤2	316 (24.6)	79 (26.2)	
2< hours ≤3	249 (19.4)	68 (22.5)	
Hours > 3	169 (13.2)	63 (20.9)	
**Sex of the child**			
Male	647 (50.4)	158 (52.3)	0.55
Female	637 (49.6)	144 (47.7)	
**Household Smoking**			
No	1103 (85.9)	245 (81.1)	0.04
Yes	181 (14.1	57 (18.9)	
**Wealth**			
Rich	544 (42.4)	97 (32.1)	<0.01
Middle	255 (19.9)	62 (20.5)	
Poor	485 (37.8)	143 (47.4)	
**Maternal Age**			
≥25 years	519 (40.4)	97 (32.1)	<0.01
<25 years	765 (59.6)	205 (67.9)	
**Birth weight**			
≥2.5 kg	1083 (84.3)	252 (83.4)	0.70
<2.5 kg	201 (15.7)	50 (16.6)	
**Parity**			
Multiparous	824 (64.2)	177 (58.6)	0.07
Nulliparous	460 (35.8)	125 (41.4)	

^a^ p-values calculated based on a 2-sided Chi-square test of independence.

Table 2 displays the results from the multivariable logistic regression models that included the main effects. Interaction terms were excluded from the final models since none of the tested interactions showed a substantial effect (exposure and smoking p = 0.06; exposure and birth weight 0 = 0.13; exposure and wealth p-0.44). The adjusted results indicated that the odds of ≥1 ALRI increased from 1.2 (95% CI: 0.7–2.2) for 1-≤2 hours to 2.1 (95% CI: 1.4–3.3) for >3 hours in front of a solid fuel cookstove (p<0.01) and with increasing poverty from 1.2 (95% CI: 0.9–1.6) among the middle group to 1.4 (95% CI: 1.2–1.7) among the poor group (p<0.001).

**Table 2:** Adjusted ORs and 95% CIs to predict children with ≥1 ALRIs.

Risk Factors	Adjusted OR^a^ (95% CI)	P-value
**Hours spent in front of polluting cookstove (per day)**		
No polluting cookstove	REF	<0.01
≤1 hours	1.2 (0.7, 2.2)	
1< hours ≤2	1.4 (1.0, 1.9)	
2< hours ≤3	1.5 (1.1, 2.0)	
Hours > 3	2.1 (1.4, 3.3)	
**Sex of the child**		
Male	REF	0.25
Female	1.1 (1.0, 1.2)	
**Household Smoking**		
No	REF	0.10
Yes	1.3 (1.0, 1.8)	
**Wealth**		
Rich	REF	<0.001
Middle	1.2 (0.9, 1.6)	
Poor	1.4 (1.2, 1.7)	
**Maternal Age**		
≥25 years	REF	0.07
<25 years	1.3 (1.0, 1.8)	
**Birth weight**		
≥2.5 kg	REF	0.60
<2.5 kg	1.1 (0.8, 1.5)	
**Parity**		
Multiparous	REF	0.14
Nulliparous	1.2 (0.9, 1.6)	

^a^ Includes all variables shown in the table.

## Discussion

In our large observational study in rural Central India, we found that after adjusting for other covariates, the odds of a child having ≥1 ALRI increases from 1.2 (95% CI: 0.7–2.2) for ≤1 hour to 2.1 (95% CI: 1.4–3.3) for >3 hours that the mother spends in front of a solid fuel cookstove (p<0.01) compared with those with no exposure. Our study showed that infants are usually inside the house when their mother is cooking, and in poorly ventilated households. another study has shown that this increases the risk for ALRI [12].

Our results are consistent with detailed and costly exposure measurements made in the RESPIRE study where Smith et al [14] found a direct exposure-response relationship between HAP and ALRIs, with increasing exposure, measured by personal carbon monoxide exposure, being associated with increased risk of ALRIs. However, as noted above, even though our results are consistent with the RESPIRE study, the results need to be confirmed in a study focused on the relation between duration of time spent cooking and the measures of personal exposure and ALRI. In Nepal, Bates et al [27] found a similar relationship between exposure to biomass cooking fuel and ALRI by measuring PM_2.5_ concentrations in household kitchens. Rylance et al [28] found that particulate exposure caused a dose-dependent increase in inflammatory cytokine production and decrease in phagocytosis of *S*. *pneumoniae* and oxidative burst, which would be consistent with HAP exposure interfering with an immune response to the pneumococcus. Our study suggests that a mother’s duration of cooking could be a simple proxy marker of exposure to household air pollutants in assessing risk of ALRI in young infants when a direct measure of the child’s exposure is not available. However, additional studies are needed to understand the complexities of measures of air quality, the actual exposure of the child to particulate matter and CO and other pollutants combined with improved assessment of the diagnosis of ALRI in relation to duration of cooking.

Our study has several strengths. First, we followed a large number of children using the Maternal and Newborn Health Registry study which has been enrolling and following women and their babies since 2009 and is run by highly trained study staff who receive on-going central and refresher trainings to ensure consistency of the data. Second, we adjusted for confounding, particularly wealth and tobacco smoking using detailed information on wealth as defined in DHS [26]. Third, we used a daily diary to improve mothers’ recall of number of days and timing of illness.

Our study also has some limitations. First, our definition of acute lower respiratory infection relied on maternal recall instead of physician diagnosis or chest x-ray. While some mis-classification of ALRI may have occurred, we expect it to be non-differential by the cookstove as ASHA workers were not trained to specifically look for ALRIs in houses for the type of stove and were also unaware of the study hypothesis. Second, use of solid fuels in the household is only a proxy of actual exposure to HAP and it would have been ideal to have actual exposure measurements to further evaluate the exposure-response relationship. Actual measurements would have also helped us to better classify households with clean cookstoves according to their true exposure level based on other sources of HAP. However, over 95% of households in our population have electricity so we feel confident that sources other than the cooking stove such as kerosene lamps are not a major contributor to HAP. Similarly, since we did not measure the child’s actual exposure to HAP, duration of mother’s cooking with a biomass stove is only a proxy of the child’s exposure and as noted above the actual exposure of the child to HAP and duration of cooking in the household needs to be studied further. Ambient air pollution data were not collected as part of this study. We did not specifically study lack of breastfeeding in our population because 99.4% of the children were exclusively breastfed during the study period. Similarly, we were not able to study maternal smoking status as over 99% of mothers reported not smoking. We also did not specifically evaluate immunization status because the conjugate pneumococcal vaccine was not widely available during the study period (only 15% of the children had received at least one of the three doses by age 6 months), but the H influenza vaccine status was up to date in 45% of the children during the study period. Kitchen characteristics were similar across our sample (75% cooked in a separate room used as a kitchen). Finally we were unable to study the effect of undernutrition because we did not conduct anthropometry during this study.

In summary, our study indicates an association between increased duration of exposure to HAP and development of ALRI in an infant under the age of 6 months. We believe that until HAP can be measured accurately, assessing a child’s exposure to HAP based on the mother’s duration of cooking using a biomass stove when the child is inside the household may be a simple way of evaluating the infant’s risk of ALRI. Until households can access clean cooking fuels, finding other ways to reduce an infant’s time indoors while a biomass stove is in use could decrease episodes of ALRI. The challenge will be finding logistically feasible ways for this to occur.

## Conclusions

The findings of our study indicate that increasing the time that a mother spends cooking over a biomass stove while her child is in the house may be associated with the child developing ALRI. Finding ways to reduce the number of hours that infants are exposed to polluting fuels during cooking time, such as educating mothers to have other household members care for the infant away from the stove while the mother is cooking could reduce the risk of ALRI in children under six months of age. This approach could provide an interim solution until all Indian households can access clean cooking fuel. How feasible this solution is in the Indian context needs to be further studied.

## Supporting information

S1 Table Distribution of hours of exposure to HAP by place of residence for women who moved to another residence for at least 30 days during her pregnancy. (DOCX)

S1 File HAP-ALRI add-on data. (CSV)

S1 Supporting Information HAP01 questionnaire in English. (PDF)

S2 Supporting Information HAP01 questionnaire in Marathi. (PDF)

S3 Supporting Information HAP02 questionnaire in English. (PDF)

S4 Supporting Information HAP02 questionnaire in Marathi. (PDF)

S5 Supporting Information HAP03 questionnaire in English. (PDF)

S6 Supporting Information HAP03 questionnaire in Marathi. (PDF)

S7 Supporting Information Pictorial diary in English. (PDF)

S8 Supporting Information Pictorial diary in Marathi. (PDF)
