# Supplementary material for: Duration of solid fuel cookstove use is associated with increased risk of acute lower respiratory infection among children under six months in rural central India
Source: PLoS One. 2019 Oct 24;14(10):e0224374. doi: 10.1371/journal.pone.0224374 (PMC6812868; doi:10.1371/journal.pone.0224374)
Supplement: S1 Table — (DOCX) [file pone.0224374.s001.docx]

**Supporting Table 1. Distribution of hours of exposure to HAP by place of residence for women who moved to another residence for at least 30 days during her pregnancy.**

|  | **Residence** | |  |
| --- | --- | --- | --- |
|  | Primary | Secondary |  |
|  | N (Col %) | | P-value^a^ |
| **Hours spent in front of polluting cookstove (per day)** | | | |
| No polluting cookstove | 29 (30.2) | 29 (30.2) | 0.41 |
| <1 hour | 11 (11.5) | 13 (13.5) |  |
| 1< hours <2 | 26 (27.1) | 22 (22.9) |  |
| 2< hours <3 | 21 (21.9) | 15 (15.6) |  |
| Hours > 3 | 9 (9.4) | 17 (17.7) |  |

^a^ p-values calculated based on a 2-sided Chi-square test of independence.
