# Supplementary material for: Duration of solid fuel cookstove use is associated with increased risk of acute lower respiratory infection among children under six months in rural central India
Source: PLoS One. 2019 Oct 24;14(10):e0224374. doi: 10.1371/journal.pone.0224374 (PMC6812868; doi:10.1371/journal.pone.0224374)
Supplement: S1 Supporting Information — (PDF) [file pone.0224374.s003.pdf]

This form should be conducted 14 days after birth (range 7-21 days) at the house where the respondent is currently residing. Where needed, put X next to correct response, otherwise complete answers as indicated.

DATE OF DELIVERY: **DATE ___ ___/ ___ ___ /___ ___**
D D M M Y Y

A. HOUSEHOLD CHARACTERISTICS

1. How many total persons usually live in your household, including the most recent birth if child is alive? **Total persons**: ______

2. How many persons that usually live in your household are children under age 5, including the most recent birth if child is alive? **Persons under 5**: ______

3. How many rooms are in your house? **Total rooms**: ______

4. Does anyone, not including yourself, smoke cigarettes/bidis or tobacco from a chillum pipe inside your house? (select ONE)
   1 |__|Yes, daily 2 |__|Yes, occasionally (less than daily) 3 |__|No, never

Observe or ask:

5. What is the main material of the floor of your house? (select ONE)
   1 |__|Natural floor
   2 |__|Rudimentary floor
   3 |__|Finished floor

6. What is the main material of the roof of your house? (select ONE)
   1 |__|Natural roof
   2 |__|Rudimentary roof
   3 |__|Finished roof

7. What is the main material of the <u>exterior</u> walls of your house? (select ONE)
   1 |__|Natural walls
   2 |__|Rudimentary walls
   3 |__|Finished walls

8. Does any member of your household have:

	YES	NO
a. A bicycle?	1 |__|	2 |__|
b. A motorcycle or motorscooter?	1 |__|	2 |__|
c. A car or truck?	1 |__|	2 |__|

9. Does your household have:

	YES	NO		YES	NO
a. Electricity?	1 |__|	2 |__|	g. A pressure cooker?	1 |__|	2 |__|
b. A radio?	1 |__|	2 |__|	h. A chair?	1 |__|	2 |__|
c. A television?	1 |__|	2 |__|	i. A table?	1 |__|	2 |__|
d. A non-mobile telephone?	1 |__|	2 |__|	j. A cot/bed?	1 |__|	2 |__|
e. A refrigerator?	1 |__|	2 |__|	k. An electric fan?	1 |__|	2 |__|
f. A mattress?	1 |__|	2 |__|			

10. How many mobile phones do members of your household own? **Total mobile phones**: ______

11. What kind of toilet facility do members of your household usually use? (select ONE)
    1 |__|Flush/pour flush to sewer system, septic tank, or pit latrine
    2 |__|Flush/pour flush to somewhere else
    3 |__|Pit latrine with slab or ventilated improved pit latrine (VIP)
    4 |__|Pit latrine without slab/open pit
    5 |__|Bucket
    6 |__|No facility, bush, field
    7 |__|Other (specify) _______________

12. What is the permanent source of drinking water for members of your household? (select ONE)
    1 |__|Piped into dwelling, yard, or plot
    2 |__|Public tap or standpipe
    3 |__|Tubewell or borehole
    4 |__|Protected well or protected spring
    5 |__|Unprotected well or unprotected spring
    6 |__|Surface water (river, pond, stream, etc.)
    7 |__|Tanker truck
    8 |__|Other (specify) _______________

	YES	NO
13. Does any member of your household own agricultural land? (if NO skip to A15)	1 |__|	2 |__|

14. How many hectares of agricultural land do members of your household own? **Hectares:** ___ ___ ___ .___

	YES	NO
15. Does your household own any livestock, herds, other farm animals, or poultry?	1 |__|	2 |__|

16. What is the annual income of your household (in Rs)? **Annual income**: ____________

B. MATERNAL CHARACTERISTICS

1. How many months during your pregnancy did you live at another residence? (If 0 or NA skip to B3) **Months**: ______ **NA** 99|__|
   If more than 0 months, fill out HAP02 form AND complete this survey

2. During which trimesters of pregnancy did you live at another residence? (select ALL THAT APPLY)
   1 |__|First trimester 2 |__|Second trimester 3 |__|Third trimester

Select answer from past observations of mother's smoking habits

3. How often during pregnancy did you smoke cigarettes/bidis or tobacco from a chillum pipe? (select ONE)
   1 |__|Daily 2 |__|Occasionally (less than daily) 3 |__|Never

C. COOKING PRACTICES – ask of the household where the interview is being held (where woman currently resides)

1. In this household, for cooking and other purposes such as boiling water, which types of stoves do you currently use? (select ALL THAT APPLY)
   1 |__|LPG stove 2 |__|Kerosene stove 3 |__|Electric stove 4 |__|Chullah 5 |__|Open fire 6 |__|Other (specify) ____________

2. Of these types of stoves which one is used most often for cooking? (select ONE) (If LPG, Kerosene, or Electric skip to C4)
   1 |__|LPG stove 2 |__|Kerosene stove 3 |__|Electric stove 4 |__|Chullah 5 |__|Open fire 6 |__|Other (specify) ____________

3. For this stove, what type of fuel does your household mainly use while cooking? (select ALL THAT APPLY)
   1 |__|Wood 2 |__|Straw/shrubs/grass 3 |__|Agricultural crop 4 |__|Animal dung 5 |__|Coal, lignite
   6 |__|Charcoal 7 |__|Other (specify) ______________

4. Where is the cooking on this stove usually done? (select ONE) (If 'Outdoors' skip to C6)
   1 |__|In the house but in a separate room used as a kitchen 2 |__|In the house with no separate room used as a kitchen
   3 |__|In a separate building or room outside of the main house 4 |__|Outdoors

5. Observe the ventilation in the cooking area for this stove. Which are present? (Show pictures and select ALL THAT APPLY)
   1 |__|Chimney 2 |__|Window 3 |__|Vent 4 |__|Door to outside 5 |__|Other (specify) ______________

6. How many years have you been using this type of stove as your main cooking stove? **Years**: ______

7. How many hours and minutes per day do you spend in front of this stove for cooking purposes while it is lit, smoldering, or on? **Hours**: ______ **Minutes**: ______

NOTE: In front of the stove is within one meter of the stove

8. Does your household have a stove separate from the stove used for cooking that is used for other household purposes such as boiling water? (If YES skip to C10) **YES** 1 |__| **NO** 2 |__|

9. How many hours and minutes per day do you spend in front of the stove for other household purposes (not cooking) while it is lit, smoldering, or on? (Skip to end) **Hours**: ______ **Minutes**: ______

NOTE: In front of the stove is within one meter of the stove

10. Which type of stove is used most often for household purposes other than cooking, such as boiling water? (select ONE) (If LPG, Kerosene, or Electric skip to C12)
1 |__|LPG stove 2 |__|Kerosene stove 3 |__|Electric stove 4 |__|Chullah 5 |__|Open fire 6 |__|Other (specify) ____________

11. For this stove, what type of fuel does your household mainly use? (select ALL THAT APPLY)
1 |__|Wood 2 |__|Straw/shrubs/grass 3 |__|Agricultural crop 4 |__|Animal dung 5 |__|Coal, lignite
6 |__|Charcoal 7 |__|Other (specify) ______________

12. Where is this stove usually used? (If 'Outdoors' skip to C14)
1 |__|In the house but in a separate room used as a kitchen 2 |__|In the house with no separate room used as a kitchen
3 |__|In a separate building or room outside of the main house 4 |__|Outdoors

13. Observe the ventilation in the area where the stove is used. Which are present? (select ALL THAT APPLY)
1 |__|Chimney 2 |__|Window 3 |__|Vent 4 |__|Door to outside 5 |__|Other (specify) ______________

14. How many years have you been using this type of stove as your main stove for household purposes other than cooking? **Years**: ______

15. How many hours and minutes per day do you spend in front of this stove while it is lit, smoldering, or on? **Hours**: _ _ __ **Minutes**: __ _

NOTE: In front of the stove is within one meter of the stove

D. FORM COMPLETION

1. Date of form completion — **DATE** ___ ___/ ___ ___ /___ ___ (D D M M Y Y)
2. Person completing form — **Name**: ___________________
3. ID of person completing form — **ID** ___ ___ ___ ___ ___ ___ ___
