# Supplementary material for: Duration of solid fuel cookstove use is associated with increased risk of acute lower respiratory infection among children under six months in rural central India
Source: PLoS One. 2019 Oct 24;14(10):e0224374. doi: 10.1371/journal.pone.0224374 (PMC6812868; doi:10.1371/journal.pone.0224374)
Supplement: S2 Supporting Information — (PDF) [file pone.0224374.s004.pdf]

ग्लोबल नेटवर्क फॉर वूमन ॲन्ड चिल्ड्रन्स हेल्थ रिसर्च	माता व नवजात शिशु आरोग्य नोंदणी<br>MNH नोंदणी क्र. |__|__|__| |__|__|__|__|__| |__|	HAP 01
पान क्र. १	दिनांक |__|__| - |__|__| - |__|__|__|__|	Version 1.1 7/10/2013

हे पत्रक, सहभागी (माता) जेथे सध्या राहत आहे तेथे प्रत्यक्ष भेट देवून प्रसुतीच्या १४ व्या दिवसाला भरावयाचे आहे.

अ. घरघुती वैशिष्ट्ये :

0 प्रसुतिचा दिनांक : |__|__| - |__|__| - |__|__|__|__|

१. अलीकडे जन्माला आलेल्या बाळाला गृहित धरुन तुमच्या कुटुंबात एकुण किती सदस्य राहतात? **एकुण सदस्य** |__|__|

२. नुकत्याच जन्माला आलेल्या बाळाला गृहित धरुन तुमच्या कुटुंबात एकुण किती मुलं ५ वर्षा खालील आहेत? **५ वर्षा खालील मुलं** |__|__|

३. तुमच्या घरात किती खोल्या आहेत? **एकुण खोल्या** |__|__|

४. तुमच्या घरात तुम्हाला वगळून कुणी धुम्रपान करतात काय (सिगारेट, बिडी, चिलम)? (एक पर्याय निवडा)

१. |__| होय, दररोज   २. |__| होय, कधीकधी (दररोज पेक्षा कमी)   ३. |__| नाही, कधीच नाही

निरिक्षण करा वा विचारा :

५. तुमच्या घरातील तळ जमीन कोणत्या सामुग्री पासुन बनविलेली आहे? (एक पर्याय निवडा)

१. |__| नैसर्गिक तळ जमीन   २. |__| अविकसीत तळ जमीन   ३. |__| पूर्ण तळ जमीन

६. तुमच्या घराचे छत मुख्यत: कुठल्या सामुग्रीने बनविलेले आहे? (एक पर्याय निवडा)

१. |__| नैसर्गिक छत   २. |__| अविकसित छत   ३. |__| पूर्ण छत

७. तुमच्या घराच्या बाहेरिल भिंती मुख्यत: कोणत्या सामुग्रीच्या आहे? (एक पर्याय निवडा)

१. |__| नैसर्गिक भिंत   २. |__| अविकसित भिंत   ३. |__| पूर्ण भिंत

८. कुटुंबातील सदस्या कडे खालील पैंकी काय उपलब्ध आहे?	होय	नाही
अ. सायकल	१. |__|	२. |__|
ब. मोटरसायकल किंवा स्कूटर	१. |__|	२. |__|
क. कार, ट्रक किंवा ट्रॅक्टर	१. |__|	२. |__|

ग्लोबल नेटवर्क फॉर वूमन ॲन्ड चिल्ड्रन्स हेल्थ रिसर्च	माता व नवजात शिशु आरोग्य नोंदणी<br>MNH नोंदणी क्र. |__|__|__| |__|__|__|__|__| |__|	HAP 01
पान क्र. २	दिनांक |__|__| - |__|__| - |__|__|__|__|	Version 1.1 7/10/2013

९. पुढील पैकी कुठल्या सोयी तुमच्या घरी आहे?

	होय	नाही		होय	नाही
अ. विज	१. |__|	२. |__|	फ. प्रेशरकुकर.	१. |__|	२. |__|
ब. रेडिओ	१. |__|	२. |__|	ग. खुर्ची	१. |__|	२. |__|
क. टेलिविजन (टि.व्ही.)	१. |__|	२. |__|	ह. टेबल	१. |__|	२. |__|
ड. टेलिफोन	१. |__|	२. |__|	य. खाट+पलंग	१. |__|	२. |__|
इ. रेफ्रीजीरेटर (फ्रिज)	१. |__|	२. |__|	ज. ईलेक्ट्रीक पंखा	१. |__|	२. |__|
ई. गादी	१. |__|	२. |__|			

१०. तुमच्या घरातील व्यक्तींकडे एकुण किती मोबाईल फोन आहेत? **एकुण मोबाईल फोन** |__|__|

११. तुमच्या कुटुंबातील व्यक्ती मुख्यत: कोणत्या प्रकारच्या शौचालय सुविधेचा वापर करतात? (एक पर्याय निवडा)

१. |__| फ्लश, सेप्टीक टँक वा पिट शौचालय
२. |__| फ्लश शौचालय विना गटारासह
३. |__| झाकणाचे पिट शौचालय, सुधारित पिट शौचालय
४. |__| विणा झाकणाचे पिट शौचालय
५. |__| बादली
६. |__| सुविधा नाही, मोकळे मैदान, झाडेझुडपे
७. |__| अन्य (स्पष्टीकरण) ____________

१२. तुमच्या कुटुंबातील सदस्याकरिता मुख्यत: पिण्याच्या पाण्याची कुठली सोय उपलब्ध आहे? (एक पर्याय निवडा)

१. |__| व्यक्तीगत नळ
२. |__| सार्वजनिक नळ
३. |__| ट्युबवेल, बोरवेल, हापसी, बोरिंग
४. |__| संरक्षित विहिर
५. |__| असंरक्षित विहिर
६. |__| पृष्ठभागावरिल पाणी (नदी, तलाव, प्रवाह इत्यादी)
७. |__| टँकर
८. |__| अन्य (स्पष्टीकरण) ____________

१३. कुटुंबातील कुठलयाही सदस्याकडे स्वत:च्या मालकीची शेतजमीन आहे काय? |__| होय |__| नाही (प्र.क्र. १५ वर जा)

१४. तुमच्या कुटुंबातील सदस्याकडे स्वत:च्या मालकीची किती एकर शेतजमीन आहे काय? **एकर** |__|__|

१५. तुमच्या कुटुंबातील सदस्याकडे स्वत:चे गुरे ढोरे, इतर पशु किंवा कुक्कुटपालनाचा व्यवसाय आहे काय? |__| होय |__| नाही

१६. तुमच्या कुटुंबाचे वार्षीक उत्पन्न किती आहे? (रुपयां मध्ये) **वार्षीक उत्पन्न** |__|__||__|__||__|

ग्लोबल नेटवर्क फॉर वूमन अँन्ड चिल्ड्रन्स हेल्थ रिसर्च	माता व नवजात शिशु आरोग्य नोंदणी<br>MNH नोंदणी क्र. |__|__|__| |__|__|__|__|__| |__|	HAP 01
पान क्र. ३	दिनांक |__|__| - |__|__| - |__|__|__|__|	Version 1.1 7/10/2013

ब. मातेची माहिती :

१. गर्भावस्थेच्या कालावधीत तुम्ही किती महिने माहेरी किंवा इतर नातेवाईकांकडे राहील्या होत्या? |__| **महिने** |__| **लागु नाही ९९**

(जर 0 महिने किंवा लागु नसल्यास प्र.क्र. ब. ३ वर जा) (जर0 महिन्या पेक्षा जास्त असल्यास HAP 02 पत्रक भरावा.)

२. गर्भावस्थेच्या कालावधीत तुम्ही कुठल्या त्रैमासिकेत तुमच्या माहेरी किंवा इतर घरी राहील्यात? (लागू असलेले सर्व पर्याय निवडा)

१. |__| प्रथम त्रैमासिकेत २. |__| व्दितीय त्रैमासिकेत ३. |__| तृतिय त्रैमासिकेत

३. तुम्ही गर्भवती असतांना किती वेळ धुम्रपान करित होत्या (सिगारेट, बिडी, चिलम) (तुमच्या मागील निरिक्षणवरु न कुठलाही एक पर्याय निवडा)

१. |__| दररोज २. |__| कधी कधी (दररोज पेक्षा कमी) ३. |__| कधीच नाही

क. स्वयंपाकाच्या पध्दती : मुलाखत जेथे घेण्यात आली तेथील कुटुंबात विचारले जाणारे प्रश्न (जेथे सहभागी (माता) सध्या राहत आहे.)

१. सध्याच्या परिस्थितीत तुमच्या घरी स्वयंपाक व इतर कामासाठी जसे पाणी तापविणे इत्यादी कामासाठी कुठल्या प्रकारच्या चुलीचा वापर करतात? (लागू असलेले सर्व पर्याय निवडा)

१. |__| एल.पी.जी. २. |__| रॉकेल स्टोव्ह ३. |__| इलेक्ट्रीक स्टोव्ह

४. |__| चुल ५. |__| ओपन फायर, दगड विटाची चुल ६. |__| अन्य (स्पष्टीकरण) ____________

२. वरिल नमुद केलेल्या चुली पैकी मुख्यत: कुठलयाप्रकाराची चुल तुम्ही स्वंयपाकासाठी वापरता? (एक पर्याय निवडा)

१. |__| एल.पी.जी. (प्र.क्र.४ वर जा) २. |__| रॉकेल स्टोव्ह (प्र.क्र.४ वर जा) ३. |__| इलेक्ट्रीक स्टोव्ह (प्र.क्र.४ वर जा)

४. |__| चुल ५. |__| ओपन फायर, दगड विटाची चुल ६. |__| अन्य (स्पष्टीकरण) ____________

३. स्वंयपाक करतांना या चुली मध्ये तुम्ही मुख्यत: कोणत्या इंधनाचा वापर करता? (लागू असलेले सर्व पर्याय निवडा)

१. |__| लाकूड २. |__| झुडुप, पेढ्या, गवत ३. |__| शेतातील सर्पण ४. |__| शेण

५. |__| दगडी कोळसा ६. |__| लाकडी कोळसा ७. |__| अन्य (स्पष्टीकरण) ____________

४. तुम्ही स्वंयपाक मुख्यत: कुठे करता? (एक पर्याय निवडा)

१. |__| घराच्या आत पण वेगळया स्वयंपाक घरात २. |__| घराच्या आत पण वेगळे स्वयंपाक घर नसलेले

३ .|__| मुख्य घराच्या बाहेर वेगळया ईमारत वा रुम मध्ये ४. |__| घरा बाहेर (प्र.क्र.६ वर जा)

५. स्वयंपाक घरातील चुलीचा धूर बाहेर निघण्याचे कुठले स्त्रोत उपलब्ध आहे यांचे निरक्षण कराव? (चित्र दाखवा व लागु असलेले सर्व पर्याय निवडा)

१. |__| चिमणी २. |__| खिडकी ३. |__| झरोखा ४. |__| घराबाहेर उघडणारे दार ५. |__| अन्य (स्पष्टीकरण) ____________

ग्लोबल नेटवर्क फॉर वूमन ॲन्ड चिल्ड्रन्स हेल्थ रिसर्च	माता व नवजात शिशु आरोग्य नोंदणी<br>MNH नोंदणी क्र. |__|__|__| |__|__|__|__|__| |__|	HAP 01
पान क्र. ४	दिनांक |__|__| - |__|__| - |__|__|__|__|	Version 1.1 7/10/2013

६. मागील किती वर्षापासून तुम्ही या चुलीचा वापर स्वयंपाकासाठी करित आहात? |__|__| **वर्ष**

७. स्वयंपाक करण्यासाठी दिवसातून किती वेळ (तास आणि मिनिटे) तुम्ही चुली जवळ घालवता? |__|__| **तास** |__|__| **मिनिटे**
(टिप:- चुली जवळून एका मिटरच्या अंतरावर)

८. स्वयंपाकाच्या चुली व्यतिरिक्त तुमच्याकडे इतर कामाकरिता जसे पाणी गरम करण्यासाठी वेगळी चुल उपलब्ध आहे काय? |__| होय |__| नाही
(जर होय तर प्र क्रं. क.१० वर जा)

९. स्वयंपाकाच्या व्यतिरिक्त इतर घरकामासाठी दिवसातून कितीवेळ (तास आणि मिनिटे) तुम्ही चुली जवळ घालवता? |__|__| **तास** |__|__| **मिनिटे**
(टिप:- चुली जवळून एका मिटरच्या अंतरावर) (**ड** वर जा)

१०. स्वयंपाकाच्या व्यतीरिक्त कुठल्या प्रकारची चूल तुम्ही पाणी गरम करण्यासाठी वापरता? (एक पर्याय निवडा)
१. |__| एल.पी.जी.(प्र.क्र.१२ वर जा) २. |__| रॉकेल स्टोव्ह(प्र.क्र.१२ वर जा) ३. |__| इलेक्ट्रीक स्टोव्ह (प्र.क्र.१२ वर जा)
४. |__| चुल ५. |__| ओपन फायर, दगड विटाची चुल ६. |__| अन्य (स्पष्टीकरण) ____________

११. या चुलीसाठी तुम्ही मुख्यत: कुठल्या प्रकारच्या इंधनाचा वापर करता? (लागू असलेले सर्व पर्याय निवडा)
१. |__| लाकूड २. |__| झुडुप, पेढ्या, गवत ३. |__| शेतातील सर्पण ४. |__| शेण
५. |__| दगडी कोळसा ६. |__| लाकडी कोळसा ७. |__| अन्य (स्पष्टीकरण) ____________

१२. हया चुलीचा वापर मुख्यत: कुठे केला जातो? (एक पर्याय निवडा)
१. |__| घराच्या आत पण वेगळया स्वयंपाक घरात २. |__| घराच्या आत पण वेगळे स्वयंपाक घर नसलेले
३. |__| मुख्य घराच्या बाहेर वेगळया ईमारत वा रुम मध्ये ४. |__| घरा बाहेर (प्र.क्र. १४ वर जा)

१३. ह्या चुलीचा धूर बाहेर निघण्याचे कुठले स्त्रोत उपलब्ध आहे यांचे निरक्षण करावे? (लागू असलेले सर्व पर्याय निवडा)
१. |__| चिमणी २. |__| खिडकी ३. |__| झरोखा ४. |__| घराबाहेर उघडणारे दार ५. |__| अन्य (स्पष्टीकरण) ____________

१४. तुम्ही अशाप्रकारची चुल गेल्या किती वर्षापासून वापरीत आहात? |__|__| **वर्ष**

१५. चुलीचा वापर होत असतांना दिवसातील किती वेळ (तास आणि मिनिटे) तुम्ही हया चुलीजवळ घालवता? |__|__| **तास** |__|__| **मिनिटे**
(टिप:- चुली जवळून एका मिटरच्या अंतरावर)

ड. संपूर्ण भरलेले नोंदणी पत्रक :

१. पत्रक पूर्ण करण्याचा दिनांक |__|__| - |__|__| - |__|__|__|__|

२. पत्रक पूर्ण करणाऱ्या व्यक्तीचे नांव ____________________ अ. ओळख क्रमांक |__|__|__|__|__|__|__|
