# Supplementary material for: Duration of solid fuel cookstove use is associated with increased risk of acute lower respiratory infection among children under six months in rural central India
Source: PLoS One. 2019 Oct 24;14(10):e0224374. doi: 10.1371/journal.pone.0224374 (PMC6812868; doi:10.1371/journal.pone.0224374)
Supplement: S3 Supporting Information — (PDF) [file pone.0224374.s005.pdf]

 SUBJECT ID ___ ___ ___ ___ ___ ___ ___ ___ ___ **DATE ___ ___/ ___ ___ /___ ___**

D D M M Y Y

This form is asking about the cooking practices and smoking habits at the residence where the woman spent time during pregnancy that is different from the house she is currently residing at. Only complete if woman answers more than 0 months to question B1 on HAP01. Where needed, put X next to correct response, otherwise complete answers as indicated.

A. COOKING PRACTICES AND SMOKING HABITS

1. In the household, for cooking and other purposes such as boiling water, which types of stoves were used? (select ALL THAT APPLY)
   1 |__|LPG stove 2 |__|Kerosene stove 3 |__|Electric stove 4 |__|Chullah 5 |__|Open fire 6 |__|Other (specify) ____________

2. Of these types of stoves which one was used most often for cooking? (select ONE) (If LPG, Kerosene, or Electric skip to A4)
   1 |__|LPG stove 2 |__|Kerosene stove 3 |__|Electric stove 4 |__|Chullah 5 |__|Open fire 6 |__|Other (specify) ____________

3. For this stove, what type of fuel did the household mainly use while cooking? (select ALL THAT APPLY)
   1 |__|Wood 2 |__|Straw/shrubs/grass 3 |__|Agricultural crop 4 |__|Animal dung 5 |__|Coal, lignite
   6 |__|Charcoal 7 |__|Other (specify) ______________

4. Where was the cooking on this stove usually done? (select ONE) (If 'Outdoors' skip to A6)
   1 |__|In the house but in a separate room used as a kitchen 2 |__|In the house with no separate room used as a kitchen
   3 |__|In a separate building or room outside of the main house 4 |__|Outdoors

5. Which forms of ventilation were present in the area where the cooking on this stove was done? (select ALL THAT APPLY)
   1 |__|Chimney 2 |__|Window 3 |__|Vent 4 |__|Door to outside 5 |__|Other (specify) ______________

6. How many hours and minutes per day did you spend in front of this stove for cooking purposes while it was lit, smoldering, or on? **Hours**: ______ **Minutes**: ______

NOTE: In front of the stove is within one meter of the stove

7. Did the household have a stove separate from the stove used for cooking that was used for other household purposes such as boiling water? (If YES skip to A9) **YES** 1 |__| **NO** 2 |__|

8. How many hours and minutes per day did you spend in front of the stove for other household purposes (not cooking) while it was lit, smoldering, or on? (Skip to A14) **Hours**: ______ **Minutes**: ______

NOTE: In front of the stove is within one meter of the stove

9. Which type of stove was used most often for household purposes other than cooking, such as boiling water? (select ONE) (If LPG, Kerosene, or Electric skip to A11)
   1 |__|LPG stove 2 |__|Kerosene stove 3 |__|Electric stove 4 |__|Chullah 5 |__|Open fire 6 |__|Other (specify) ____________

10. For this stove, what type of fuel did the household <u>mainly</u> use? (select ALL THAT APPLY)
    1 |__|Wood 2 |__|Straw/shrubs/grass 3 |__|Agricultural crop 4 |__|Animal dung 5 |__|Coal, lignite
    6 |__|Charcoal 7 |__|Other (specify) _______________

11. Where was this stove usually used? (select ONE) (If 'Outdoors' skip to A13)
    1 |__|In the house but in a separate room used as a kitchen 2 |__|In the house with no separate room used as a kitchen
    3 |__|In a separate building or room outside of the main house 4 |__|Outdoors

12. Which forms of ventilation were present in the area where this stove was used? (select ALL THAT APPLY)
    1 |__|Chimney 2 |__|Window 3 |__|Vent 4 |__|Door to outside 5 |__|Other (specify) _______________

13. How many hours and minutes per day did you spend in front of this stove while it was lit, smoldering, or on? **Hours**: ______ **Minutes**: ______

NOTE: In front of the stove is within one meter of the stove

14. Did anyone, not including yourself, smoke cigarettes/bidis or tobacco from a chillum pipe inside the house? (select ONE)
    1 |__|Yes, daily 2 |__|Yes, occasionally (less than daily) 3 |__|No, never

B. FORM COMPLETION

1. Date of form completion **DATE** ___ ___/ ___ ___ /___ ___
   D D M M Y Y
2. Person completing form **Name**: ___________________
3. ID of person completing form **ID** ___ ___ ___ ___ ___ ___ ___
