# Supplementary material for: Duration of solid fuel cookstove use is associated with increased risk of acute lower respiratory infection among children under six months in rural central India
Source: PLoS One. 2019 Oct 24;14(10):e0224374. doi: 10.1371/journal.pone.0224374 (PMC6812868; doi:10.1371/journal.pone.0224374)
Supplement: S4 Supporting Information — (PDF) [file pone.0224374.s006.pdf]

ग्लोबल नेटवर्क फॉर वूमन ॲन्ड चिल्ड्रन्स हेल्थ रिसर्च	माता व नवजात शिशु आरोग्य नोंदणी<br>MNH नोंदणी क्र. |__|__|__| |__|__|__|__|__| |__|	HAP 02
पान क्र. १	दिनांक |__|__| – |__|__| – |__|__|__|__|	Version 1.1 7/10/2013

सहभागीस (मातेला) स्वयंपाकाच्या पद्धती व धुम्रपान विषयी विचारले जाणारे प्रश्न. हे पत्रक, तेव्हा भरायचे आहे जेव्हा सहभागी (माता) HAP 01 **प्रश्न क्र. क-१** चे उत्तर ० महिने पेक्षा जास्त देयील (गर्भावस्थेच्या कालावधीत जर सहभागी (माता) माहेरी किंवा इतर नातेवाईकांकडे १ किंवा जास्त महिने राहिली असल्यास हे पत्रक तेथील घरातील माहिती बद्दल भरावे).

क. स्वयंपाकाच्या पध्दती आणि धुम्रपानाच्या सवयी :

0 प्रसुतिचा दिनांक: |__|__| – |__|__| – |__|__|__|__|

१. तुमच्या घरी स्वयंपाक व इतर कामासाठी जसे पाणी तापविणे इत्यादी कामासाठी कुठल्या प्रकारच्या चुलीचा वापर होत होता?
(लागू असलेले सर्व पर्याय निवडा)
१. |__| एल.पी.जी. २. |__| रॉकेल स्टोव्ह ३. |__| इलेक्ट्रीक स्टोव्ह
४. |__| चुल ५. |__| ओपन फायर, दगड विटाची चुल ६. |__| अन्य (स्पष्टीकरण) ________

२. वरिल नमुद केलेल्या चुली पैकी मुख्यत: कुठल्या प्रकारच्या चुलीचा वापर स्वयंपाकासाठी होत होता? (एक पर्याय निवडा)
१. |__| एल.पी.जी. (प्र.क्र.४ वर जा) २. |__| रॉकेल स्टोव्ह (प्र.क्र.४ वर जा) ३. |__| इलेक्ट्रीक स्टोव्ह (प्र.क्र.४ वर जा)
४. |__| चुल ५. |__| ओपन फायर, दगड विटाची चुल ६. |__| अन्य (स्पष्टीकरण) ________

३. स्वयंपाक करतांना या चुली मध्ये मुख्यत: कोणत्या इंधनाचा वापर होत होता? (लागू असलेले सर्व पर्याय निवडा)
१. |__| लाकूड २. |__| झुडुप, पेंढ्या, गवत ३. |__| शेतातील सर्पण ४. |__| शेण
५. |__| दगडी कोळसा ६. |__| लाकडी कोळसा ७. |__| अन्य (स्पष्टीकरण) ________

४. स्वयंपाक मुख्यत: कुठे होत होता (एक पर्याय निवडा)
१. |__| घराच्या आत पण वेगळया स्वयंपाक घरात २. |__| घराच्या आत पण वेगळे स्वयंपाक घर नसलेले
३ .|__| मुख्य घराच्या बाहेर वेगळया ईमारत वा रुम मध्ये ४. |__| घरा बाहेर (प्र.क्र ६ वर जा)

५. स्वयंपाक घरातील चुलीचा धूर बाहेर निघण्याचे कुठले स्त्रोत उपलब्ध होते? (चित्र दाखवा व लागु असलेले सर्व पर्याय निवडा)
१. |__| चिमणी २. |__| खिडकी ३. |__| झरोखा ४. |__| घराबाहेर उघडणारे दार ५. |__| अन्य (स्पष्टीकरण) ________

६. स्वयंपाक करण्यासाठी दिवसातून किती वेळ (तास आणि मिनिटे) तुम्ही चुली जवळ घालवित होतात? |__|__| **तास** |__|__| **मिनिटे**
(टिप:– चुली जवळून एका मिटरच्या अंतरावर)

७. स्वयंपाकाच्या चुली व्यतिरिक्त तुमच्याकडे इतर कामाकरिता जसे पाणी गरम करण्यासाठी वेगळी चुल उपलब्ध होती काय? |__| होय |__| नाही
(जर होय तर प्र क्रं. क.९ वर जा)

ग्लोबल नेटवर्क फॉर वूमन ॲन्ड चिल्ड्रन्स हेल्थ रिसर्च

माता व नवजात शिशु आरोग्य नोंदणी

MNH नोंदणी क्र. |__|__|__| |__|__|__|__|__| |__|

HAP 02

पान क्र. २

दिनांक |__|__| - |__|__| - |__|__|__|__|

Version 1.1 7/10/2013

८. स्वयंपाकाच्या व्यतिरिक्त इतर घरकामासाठी दिवसातून कितीवेळ (तास आणि मिनिटे) तुम्ही चुली जवळ घालवित होतात?|__|__| **तास** |__|__| **मिनिटे**
(टिप:- चुली जवळून एका मिटरच्या अंतरावर) (प्र.क्र.१४ वर जा)

९. स्वयंपाकाच्या व्यतीरिक्त कुठल्या प्रकारची चुल तुम्ही पाणी गरम करण्यासाठी वापरत होतात? (एक पर्याय निवडा)
१. |__| एल.पी.जी.(प्र.क्र.११ वर जा) २. |__| रॉकेल स्टोव्ह(प्र.क्र.११ वर जा) ३. |__| इलेक्ट्रीक स्टोव्ह (प्र.क्र.११ वर जा)
४. |__| चुल ५. |__| ओपन फायर, दगड विटाची चुल ६. |__| अन्य (स्पष्टीकरण) ____________

१०. या चुलीसाठी मुख्यत: कुठल्या प्रकारच्या इंधनाचा वापर होत होता? (लागू असलेले सर्व पर्याय निवडा)
१. |__| लाकूड २. |__| झुडुप, पेंढ्या, गवत ३. |__| शेतातील सर्पण ४. |__| शेण
५. |__| दगडी कोळसा ६. |__| लाकडी कोळसा ७. |__| अन्य (स्पष्टीकरण) ____________

११. या चुलीचा वापर मुख्यत: कुठे होत होता? (एक पर्याय निवडा)
१. |__| घराच्या आत पण वेगळया स्वयंपाक घरात २. |__| घराच्या आत पण वेगळे स्वयंपाक घर नसलेले
३. |__| मुख्य घराच्या बाहेर वेगळया ईमारत वा रुम मध्ये ४. |__| घरा बाहेर (प्र.क्र. १३ वर जा)

१२. ह्या चुलीचा धूर बाहेर निघण्याचे कुठले स्त्रोत उपलब्ध होते? (लागू असलेले सर्व पर्याय निवडा)
१. |__| चिमणी २. |__| खिडकी ३. |__| झरोखा ४. |__|घराबाहेर उघडणारे दार ५. |__| अन्य (स्पष्टीकरण) ____________

१३. चुलीचा वापर होत असतांना दिवसातील किती वेळ (तास आणि मिनिटे) तुम्ही हया चुलीजवळ घालवित होतात?**तास** |__|__| **मिनिटे** |__|__|
(टिप:- चुली जवळून एका मिटरच्या अंतरावर)

१४. तुमच्या घरात तुम्हाला वगळुन कोणी धुम्रपान (सिगारेट, बिडी, चिलम) करीत होते काय?
१. |__| होय, दररोज २. |__| कधीकधी, दररोज पेक्षा कमी ३. |__| नाही, कधीच नाही

ड. संपूर्ण भरलेले नोंदणी पत्रक :

१. पत्रक पूर्ण करण्याचा दिनांक |__|__| - |__|__| - |__|__|__|__|

२. पत्रक पूर्ण करणाऱ्या व्यक्तीचे नांव ____________________ अ. ओळख क्रमांक |__|__|__|__|__|__|__|
