# Supplementary material for: Duration of solid fuel cookstove use is associated with increased risk of acute lower respiratory infection among children under six months in rural central India
Source: PLoS One. 2019 Oct 24;14(10):e0224374. doi: 10.1371/journal.pone.0224374 (PMC6812868; doi:10.1371/journal.pone.0224374)
Supplement: S8 Supporting Information — (PDF) [file pone.0224374.s010.pdf]

*Lata Medical Research Foundation, Nagpur*

Global Network – Household Air Pollution

मातेचे नाव – ______________________

एम.एन.एच.आयडी – |__|__|__|__|__|__|__|__|__|__| प्रसुतीचा दिनांक – / / भेट क्रं..

आशाचे नाव – ______________________ माहीती घेण्याचा कालावधी– दिनांक – / / - दिनांक / /

खालीलपैकींल लक्षणे असल्यास योग्य त्या चित्रासमोर, ज्या दिवशी लक्षणे असेल त्या दिवसाच्या डब्यात √ मार्क करा किंवा टिकली लावावी.

| | | | | | | | | | | | | | | | |
|---|---|---|---|---|---|---|---|---|---|---|---|---|---|---|---|
| 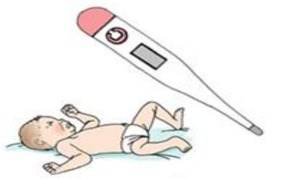 | ताप | | | | | | | | | | | | | | |
| 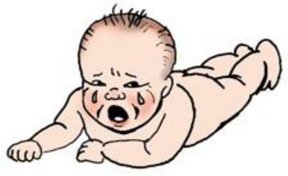 | खोकला | | | | | | | | | | | | | | |
| 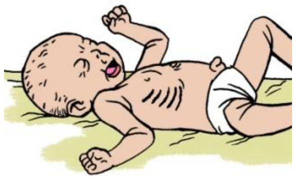 | श्वास घेण्यास त्रास | | | | | | | | | | | | | | |
| 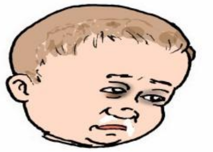 | सर्दी | | | | | | | | | | | | | | |
| 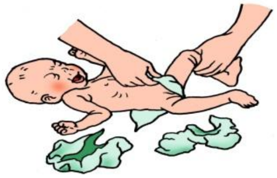 | अतीसार | | | | | | | | | | | | | | |
| 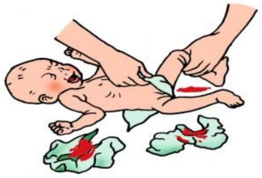 | शौचात रक्त | | | | | | | | | | | | | | |
