# Supplementary material for: Duration of solid fuel cookstove use is associated with increased risk of acute lower respiratory infection among children under six months in rural central India
Source: PLoS One. 2019 Oct 24;14(10):e0224374. doi: 10.1371/journal.pone.0224374 (PMC6812868; doi:10.1371/journal.pone.0224374)
Supplement: S6 Supporting Information — (PDF) [file pone.0224374.s008.pdf]

ग्लोबल नेटवर्क फॉर वूमन ॲन्ड चिल्ड्रन्स हेल्थ रिसर्च	माता व नवजात शिशु आरोग्य नोंदणी<br>MNH नोंदणी क्र: |__|__|__| |__|__|__|__|__| |__|	HAP 03
पान क्र: 1	दिनांक |__|__| - |__|__| - |__|__|__|__|	Version 1.1 7/10/2013

हे पत्रक दर दोन आठवड्यांनी भरावयाचे आहे. प्रथम हे पत्रक प्रसुतीच्या १४ व्या दिवसाला भरावयाचे आहे व तदनंतर दर दोन आठवड्यांनी या प्रकारे ६ महिन्यापर्यंत भरावयाचे आहे.

अ. मृत्युची माहिती :

०. **प्रसुतिचा दिनांक** : |__|__| - |__|__| - |__|__|__|__| **०० भेट क्र :** |__|__|

१. बाळ जिवंत आहे काय? (जर होय तर प्र.क्रं. ब १ वर जा, जर नाही तर फक्त विभाग अ भरा) |__| होय |__| नाही

२. बाळ मृत पावले असल्यास मृत्युचा दिनांक |__|__| - |__|__| - |__|__|__|__|

३. बाळ मृत असल्यास आईच्या म्हणण्याप्रमाणे, बाळाच्या मृत्युचे काय कारण होते? (लागू असलेले सर्व पर्याय निवडा)

१. |__| निमोनिया २. |__| अतिसार ३. |__| इनसेफेलाईटीस (मेदूला आलेली सूज)

४. |__| तापाचा आजार ५. |__| अन्य (स्पष्टीकरण)

ब. अस्वास्थता :

		होय	नाही	माहित नाही
१.	मागील दोन आठवड्या मध्ये तुमचे बाळ कधी तापाने आजारी होते काय?<br>(जर नाही किंवा माहिती नाहीं तर प्र.क्र. **ब. ३** वर जा)	१. |__|	२. |__|	३. |__|
२.	मागील दोन आठवड्या मध्ये तुमच्या बाळाला किती दिवस ताप होता?			एकुण दिवस |__|__|
३.	मागील दोन आठवड्यामध्ये तुमच्या बाळाला कधी खोकला झाला होता काय?<br>(जर नाही किंवा माहिती नाहीं तर प्र. क्र. **ब.७** वर जा)	१. |__|	२. |__|	३. |__|
४.	मागील दोन आठवड्यामध्ये तुमच्या बाळाला किती दिवस खोकला होता?			एकुण दिवस |__|__|
५.	जेव्हा तुमचे बाळ खोकल्याने आजारी होते तेव्हा तो/ती नेहमी पेक्षा जास्त गतीने श्वास घेत होते काय किंवा श्वास घ्यायला त्रास होता काय?<br>(टिप : श्वास घेण्यास त्रास म्हणजे श्वास घेताना आवाज येणे किंवा श्वासाची गती जास्त, असल्याने स्तनपानास त्रास होणे)	१. |__|	२. |__|	३. |__|

६. जेव्हा तुमच्या बाळाला खोकला होता तेव्हा कुठल्याप्रकारची औषधे दिलीत? (लागू असलेले सर्व पर्याय निवडा व उपलब्ध असल्यास, औषधाच्या बाटलीचे नाव तपासा) ---------------------------

१. |__|काही नाही २|__| प्रतिजैविक ३.|__|मलेरियाची औषधी ४. |__| पॅरासिटामोल (ताप कमी करण्यासाठी) ५.|__|अन्य (स्पष्टीकरण) -----

ब औषधींचे नाव ____________________________

क बाळाला किती दिवस औषधे दिली |__|__| दिवस

ग्लोबल नेटवर्क फॉर वूमन ॲन्ड चिल्ड्रन्स हेल्थ रिसर्च	माता व नवजात शिशु आरोग्य नोंदणी<br>MNH नोंदणी क्र: |__|__|__| |__|__|__|__|__| |__|	HAP 03
पान क्र: 2	दिनांक |__|__| - |__|__| - |__|__|__|__|	Version 1.1 7/10/2013

	होय	नाही	माहित नाही
७. मागील दोन आठवड्या मध्ये तुमच्या बाळाला सर्दी होती काय ? (नाकातुन पाणी निघणे वा नाक बंद झाल्यासारखे वाटणे) (जर नाही किंवा माहिती नाही तर प्र.क्र.**ब. ९** वर जा)	१. |__|	२. |__|	३. |__|
८. मागील दोन आठवड्या मध्ये तुमच्या बाळाला किती दिवस सर्दी होती? (नाकातून पाणी निघणे वा नाक बंद झाल्यासारखे वाटणे)			एकुण दिवस |__|__|
९. मागील दोन आठवड्या मध्ये तुमच्या बाळाला कधी अतिसार झाला होता काय? (नेहमीच्या शौचापेक्षा वेगळ्या प्रकारच्या शौच होणे जसे तिन वेळापेक्षा जास्त पातळ शौच होणे) (जर नाही किंवा माहिती नाही तर प्र.क्र. **क.१** वर जा)	१. |__|	२. |__|	३. |__|
१०. मागील दोन आठवड्या मध्ये तुमच्या बाळाला किती दिवस अतिसार होता?			एकुण दिवस |__|__|
११. तुमच्या बाळाच्या शौचामध्ये रक्त असल्याचे तुम्हाला आढळले काय?	१. |__|	२. |__|	३. |__|
१२. आपण आपल्या बाळाला अतिसार प्रारंभ झाल्यापासुन खालील पैकी पेय पिण्यास दिले होते काय?			
अ. पातळ पदार्थांचे विशेष पॅकेट (ORS)	१. |__|	२. |__|	३. |__|
ब. सरकारी मान्यताप्राप्त घरघुती द्रवपदार्थ (साखर, मिठ व पाणी चे घोळ)	१. |__|	२. |__|	३. |__|

क. स्तनपान :

	होय	नाही
१. तुम्ही तुमच्या बाळाला स्तनपान देत आहात का? (जर नाही तर प्रश्न क्र. **ड. १** वर जा)	१. |__|	२. |__|
२. तुम्ही तुमच्या बाळाला निव्वळ स्तनपान देत आहात काय?	१. |__|	२. |__|

टिप : नवजात शिशुला स्तनपान (मागील २४ तासात दिलेले आईचे दुध, त्यक्त दुध किंवा मुलाला अंगावर पाजणारी दाई द्वारे). तसेच ORS, जीवनसत्व किंवा खनिज यांचे थेंब आणि औषधे व्यतिरिक्त काहीच नाही, पाणी सुद्धा न देणे.

ग्लोबल नेटवर्क फॉर वूमन ॲन्ड चिल्ड्रन्स हेल्थ रिसर्च	माता व नवजात शिशु आरोग्य नोंदणी<br>MNH नोंदणी क्र: |__|__|__| |__|__|__|__|__| |__|	HAP 03
पान क्र: 3	दिनांक |__|__| - |__|__| - |__|__|__|__|	Version 1.1 7/10/2013

ड. बाळाची स्थिती :

मुलाखतीच्या आधल्या दिवशी, घरातील चुलीचा वापर होत असतांना बाळाचे स्थान (बाळ कुठे होते) जाणून घेणे हा ह्या विभागाचा उद्देश आहे. (चुलीचा वापर होणे म्हणजेच, चुल पेटत राहणे, धुर निघणे किंवा उपयोगात असणे)

खालील प्रश्न सर्व प्रकारच्या चुलीसाठी लागू राहतील जसे चुल, एल.पी.जी. ओपन फायर/दगड विटांची चुल इत्यादी.

सकाळ : उठल्यापासून तर १२ वाजेपर्यंत **दुपार :** १२ पासून तर ४ वाजेपर्यंत **सायंकाळ :** ४ वाजेपासून झोपेपर्यंत

अ. पुढील नमूद केलेल्या वेळेपैकी कुठल्या वेळेला तुमच्या कडील चुल वापरल्या जाते? (जर नाही तर पुढील वेळेसाठी तपासा)

ब. चुल चालु असतांनी तुमचे बाळ पुढील नमुद केलेल्या वेळी आत होते किंवा बाहेर होते?

क. पुढील नमुद केलेल्या वेळेपैकी कुठल्या वेळी तुमच्या बाळ चुली जवळ १ मिटरच्या अंतरावर होते वा १ मिटर पेक्षा जास्त अंतरावर होते?

ड. पुढील नमुद केलेल्या वेळेपैकी तुमच्याकडे कोणत्या प्रकारची चुल वापरण्यात आली? प्राथमिक चुल, दुय्यम चुल (इतर घरघुती उपयोगासाठी जसे पाणी तापविण्यासाठी) किंवा दोन्ही चुल. (जर घरी एकच चुल असेल तर प्राथमिक निवड)

वेळ	अ. चुलीचा वापर		ब. बाळाचे स्थान		क. चुलीपासुनचे अंतर		ड. चुलीचा प्रकार		
	होय	नाही	आत	बाहेर	चुलीच्या जवळ	चुलीच्या दुर	प्राथमिक	दुय्यम	दोन्ही
सकाळ	१. |__|	२. |__|	१. |__|	२. |__|	१. |__|	२. |__|	१.|__|	२.|__|	३.|__|
दुपार	१. |__|	२. |__|	१. |__|	२. |__|	१. |__|	२. |__|	१.|__|	२.|__|	३.|__|
सायंकाळ	१. |__|	२. |__|	१. |__|	२. |__|	१. |__|	२. |__|	१.|__|	२.|__|	३.|__|

ग्लोबल नेटवर्क फॉर वूमन ॲन्ड चिल्ड्रन्स हेल्थ रिसर्च	माता व नवजात शिशु आरोग्य नोंदणी<br>MNH नोंदणी क्र: |__|__|__| |__|__|__|__|__| |__|	HAP 03
पान क्र: 4	दिनांक |__|__| - |__|__| - |__|__|__|__|	Version 1.1 7/10/2013

इ. लसीकरण, मानवमीती आणि धुम्रपान : (हा विभाग फक्त सहा महिन्याच्या भेटीच्या वेळी भरावा)

१. खालील नमुद केलेल्या लसी पैकी तुमच्या बाळाला कुठली लस दिल्या गेलेली आहे.

लस	डोज	पुर्ण	अपुर्ण	नाही
BCG	(१ डोज)	१. |__|	२. |__|	३. |__|
Hep B	(३ डोज)	१. |__|	२. |__|	३. |__|
OPV	(३ डोज किंवा ३ IPV डोज)	१. |__|	२. |__|	३. |__|
DPT	(३ डोज)	१. |__|	२. |__|	३. |__|
Hib	(३ डोज)	१. |__|	२. |__|	३. |__|
Pneumococcal	(३ डोज)	१. |__|	२. |__|	३. |__|
Rota Virus	(२ डोज Rotarix किंवा ३ डोज Rotateg)	१. |__|	२. |__|	३. |__|

२.	वजन	|__|__|__| किग्रॅ	९९७ |__| उपस्थित नाही	९९८ |__| नाकारले	९९९ |__| इतर
३.	उंची	|__|__|__| सेमी	९९७ |__| उपस्थित नाही	९९८ |__| नाकारले	९९९ |__| इतर
४.	डोक्याचे परिघ	|__|__|__| सेमी	९९७ |__| उपस्थित नाही	९९८ |__| नाकारले	९९९ |__| इतर
५.	दंडाचे परिघ	|__|__|__| सेमी	९९७ |__| उपस्थित नाही	९९८ |__| नाकारले	९९९ |__| इतर

६. बाळाच्या जन्मापासून तुम्ही कितीदा धुम्रपान करता (सिगारेट/बिडी/चिलम)? (मागील ६ महिन्याच्या माहिती व निरीक्षणावरुन उत्तराचा एक पर्याय निवडा)

१. |__| रोज   २. |__| कधी कधी (रोज पेक्षा कमी)   ३. |__| कधीच नाही.

फ. संपूर्ण भरलेले नोंदणी पत्रक :

१. पत्रक पूर्ण करण्याचा दिनांक |__|__| - |__|__| - |__|__|__|__|

२. पत्रक पूर्ण करणाऱ्या व्यक्तीचे नाव ___________________________   अ. ओळख क्रमांक|__|__|__|__|__|__|__|
