# Supplementary material for: Duration of solid fuel cookstove use is associated with increased risk of acute lower respiratory infection among children under six months in rural central India
Source: PLoS One. 2019 Oct 24;14(10):e0224374. doi: 10.1371/journal.pone.0224374 (PMC6812868; doi:10.1371/journal.pone.0224374)
Supplement: S7 Supporting Information — (PDF) [file pone.0224374.s009.pdf]

Lata Medical Research Foundation, Nagpur

Global Network – Household Air Pollution

Name of Participant –

MNH ID – I___I___I___I___I___I___I___I___I___I___I   Date of Delivery – / /   Visit No.

Name of ASHA worker –   Duration of follow up – Date / / - Date / /

Place [√] mark or **Bindi** in appropriate box, if any of the following signs are present.

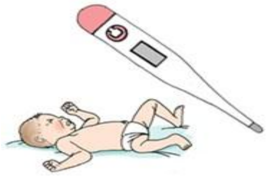	Fever	Wk 1							Wk 3						
		Wk 2							Wk 4						
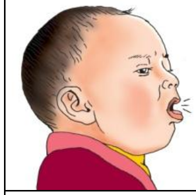	Cough	Wk 1							Wk 3						
		Wk 2							Wk 4						
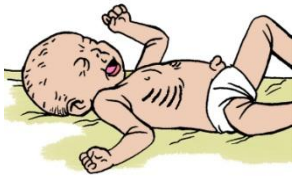	Difficult breathing	Wk 1							Wk 3						
		Wk 2							Wk 4						
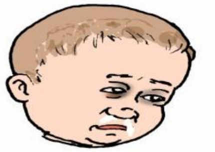	Runny nose	Wk 1							Wk 3						
		Wk 2							Wk 4						
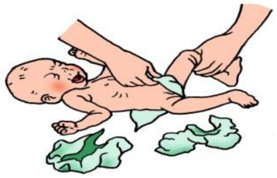	Diarrhea	Wk 1							Wk 3						
		Wk 2							Wk 4						
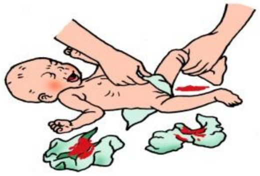	Blood in stool	Wk 1							Wk 3						
		Wk 2							Wk 4						
