# Supplementary material for: Duration of solid fuel cookstove use is associated with increased risk of acute lower respiratory infection among children under six months in rural central India
Source: PLoS One. 2019 Oct 24;14(10):e0224374. doi: 10.1371/journal.pone.0224374 (PMC6812868; doi:10.1371/journal.pone.0224374)
Supplement: S5 Supporting Information — (PDF) [file pone.0224374.s007.pdf]

 SUBJECT ID ___ ___ ___ ___ ___ ___ ___ ___ ___ **DATE ___ ___/ ___ ___ /___ ___** (D D M M Y Y)

This form should be conducted every 2 weeks (+/- 3 days) starting 14 days post delivery until 6 months post delivery. Where needed, put X next to correct response, otherwise complete answers as indicated. Other Comments are same as given in HAP01.

DATE OF DELIVERY: ___ ___/ ___ ___ /___ ___ (D D M M Y Y) **VISIT NO. |__|__|**

A. MORTALITY

1. Is child alive? (If YES then skip to B1; if NO complete section A and skip to end) — **YES** 1 |__| **NO** 2 |__|

2. What was the child's date of death? **Date of death ___ ___/ ___ ___ /___ ___** (D D M M Y Y)

3. According to the mother, what was the most likely cause of death of the child? (select ALL THAT APPLY)
   1 |__|Pneumonia 2 |__|Diarrhea 3 |__|Encephalitis 4 |__|Febrile illness 5 |__|Other (specify) _______________

B. MORBIDITY

1. Has (NAME) been ill with a fever at any time in the last 2 weeks? (If NO or DK skip to B3) — **YES** 1 |__| **NO** 2 |__| **DK** 3 |__|

2. How many days did (NAME) have a fever in the last 2 weeks? **Number of days: ______**

3. Has (NAME) had an illness with a cough at any time in the last 2 weeks? (If NO or DK skip to B6) — **YES** 1 |__| **NO** 2 |__| **DK** 3 |__|

4. How many days did (NAME) have a cough in the last 2 weeks? **Number of days: ______**

5. When (NAME) had an illness with a cough, did he/she breathe faster than usual with short, rapid breaths or have difficulty breathing? — **YES** 1 |__| **NO** 2 |__| **DK** 3 |__|

NOTE: Difficulty breathing is defined as grunting or difficulty feeding because of fast breathing

5a. How many days did (NAME) have a fast breathing in the last 2 weeks? **Number of days: ______**

6. What treatments was (NAME) given since he/she started having a cough and/or fever? (select ALL THAT APPLY)
   Look at the bottles to verify, if available.
   a. 1 |__|Nothing 2 |__|Antibiotics 3 |__|Antimalarials 4 |__|Fever reducer (such as paracetamol) 5 |__|Other (specify) ___________

b. Name of medication(s):___________________________________
c. How many days did (NAME) take the medication? **Number of days:** ______

		**YES**	**NO**	**DK**
7.	Did (NAME) have a blocked or runny nose in the last 2 weeks? (If NO or DK skip to B9)	1 |__|	2 |__|	3 |__|

8. How many days did (NAME) have a blocked or runny nose in the last 2 weeks? **Number of days:** ______

		**YES**	**NO**	**DK**
9.	In the last 2 weeks, has (NAME) had diarrhea, defined as 3 or more unformed stools within one day that are different from the normal? (If NO or DK skip to C1)	1 |__|	2 |__|	3 |__|

10. How many days did (NAME) have diarrhea in the last 2 weeks? **Number of days:** ______

		**YES**	**NO**	**DK**
11.	Was there any blood or mucus in the stools?	1 |__|	2 |__|	3 |__|

12. Was (NAME) given any of the following to drink at any time since he/she started having the diarrhea?

		**YES**	**NO**	**DK**
a.	A fluid made from a special packet called ORS	1 |__|	2 |__|	3 |__|
b.	A government-recommended homemade fluid	1 |__|	2 |__|	3 |__|

C. BREASTFEEDING

		**YES**	**NO**
1.	Are you breastfeeding (NAME)? (If NO skip to D1)	1 |__|	2 |__|
2.	Are you exclusively breastfeeding (NAME)?	1 |__|	2 |__|

NOTE: Exclusively breastfeeding is defined as feeding the infant with breast milk (either directly from mother's breast, expressed milk, or by a wet nurse) within last 24 hours. Also allows the infant to feed with ORS, drops or syrups of vitamins, minerals and medicines and **NOTHING ELSE**, including water.

CONTINUE ONTO PAGE 3

 SUBJECT ID ___ ___ ___ ___ ___ ___ ___ ___ ___ **DATE ___ ___/ ___ ___ /___ ___** (D D M M Y Y)

D. CHILD LOCATION

This section tracks the child's location while a household stove was in use at different times during the day prior to interview. A stove was in use if it was burning, smoldering, lit, or on. This refers to any type of stove: chullah, LPG, open fire, etc. Morning is from waking – 12 noon; Afternoon is 12 noon – 4 PM; Evening is 4 PM – bed time.

a. Was any stove in use at your household at any point during the following times of day? (If NO skip to next time of day)
b. Did (NAME) spend more time inside **OR** outside of the house while the stove was in use?
c. Was (NAME) near (within 1 meter) **OR** far (more than 1 meter) from the stove at any point while it was in use?
d. Was the stove that was in use the primary cooking stove, the secondary stove used for other household purposes such as boiling water, or both stoves? (If household only has one stove then select primary)

Primary Stove is the stove the household most often uses for cooking food
Secondary Stove is the stove other than the primary stove that may be used for other household purposes such as boiling water

TIMES OF DAY	STOVE IN USE YES	NO	INSIDE	OUTSIDE	NEAR STOVE	FAR FROM STOVE	PRIMARY STOVE	SECONDARY STOVE	BOTH STOVES
1. **MORNING**	a. 1 |__|	2 |__|	b. 1 |__|	2 |__|	c. 1 |__|	2 |__|	d. 1 |__|	2 |__|	3 |__|
2. **AFTERNOON**	a. 1 |__|	2 |__|	b. 1 |__|	2 |__|	c. 1 |__|	2 |__|	d. 1 |__|	2 |__|	3 |__|
3. **EVENING**	a. 1 |__|	2 |__|	b. 1 |__|	2 |__|	c. 1 |__|	2 |__|	d. 1 |__|	2 |__|	3 |__|

E. 6 MONTH IMMUNIZATIONS, ANTHROPOMETRY, AND MATERNAL SMOKING – only complete at 6 month visit

1. Did (NAME) receive the following vaccinations in full, partially, or not at all:

	Vaccine	Doses	FULL	PARTIAL	NONE
a.	BCG	(1 dose)	1 |__|		3 |__|
b.	Hep B	(3 doses)	1 |__|	2 |__|	3 |__|
c.	OPV	(3 doses, or 3 IPV doses)	1 |__|	2 |__|	3 |__|
d.	DPT	(3 doses)	1 |__|	2 |__|	3 |__|
e.	Hib	(3 doses)	1 |__|	2 |__|	3 |__|
f.	Pneumococcal	(3 doses)	1 |__|	2 |__|	3 |__|
**g.**	Rotavirus	(2 doses Rotarix or 3 doses Rotateq)	1 |__|	2 |__|	3 |__|

CONTINUE ONTO PAGE 4

2. Weight in kilograms ___ ___ .___ ___ kg 997 |__|Not present 998 |__|Refused 999 |__|Other _________
3. Height in centimeters ___ ___ ___ . ___ cm 997 |__|Not present 998 |__|Refused 999 |__|Other _________
4. Head circumference in centimeters ___ ___ . ___ cm 997 |__|Not present 998 |__|Refused 999 |__|Other _________
5. Upper arm circumference in centimeters___ ___ . ___ cm 997 |__|Not present 998 |__|Refused 999 |__|Other _________

Select answer from observations of mother's smoking habits during the past 6 months

6. Since (NAME) was born, how often do you smoke cigarettes/bidis or tobacco from a chillum pipe? (select ONE)
   1 |__|Daily 2 |__|Occasionally (less than daily) 3 |__|Never

F. FORM COMPLETION

1. Date of form completion **DATE** ___ ___/ ___ ___ /___ ___ (D D M M Y Y)
2. Person completing form **Name**: __________________
3. ID of person completing form **ID** ___ ___ ___ ___ ___ ___ ___
